# Experimental test of the collapse time of a delocalized photon state

**DOI:** 10.1038/s41598-019-48387-8

**Published:** 2019-08-15

**Authors:** Francesco Garrisi, Micol Previde Massara, Alberto Zambianchi, Matteo Galli, Daniele Bajoni, Alberto Rimini, Oreste Nicrosini

**Affiliations:** 1 Università degli Studi di Pavia, Dipartimento di Fisica, 27100 Pavia, via A. Bassi 6, Italy; 2 Università degli Studi di Pavia, Dipartimento di Ingegneria Industriale e dell’Informazione, 27100 Pavia, via Ferrata 5, Italy; 3 Istituto Nazionale Fisica Nucleare - Sezione di Pavia, 27100 Pavia, via A. Bassi 6, Italy

**Keywords:** Quantum optics, Single photons and quantum effects

## Abstract

We investigate whether the collapse of the quantum state of a single photon split between two space-like separated places takes a nonvanishing time. We realize this by using a source of heralded single photons, then splitting the resulting single photon state and letting it propagate over distances much larger than the experimental time resolution times the speed of light *c*. We find no additional delay within our accuracy and set a lower limit for the speed of collapse of the quantum state to 1550*c*.

## Introduction

In the standard formulation of Quantum Mechanics two principles of evolution of the state vector of a given system are assumed. According to the first one, when the system is closed it evolves in time according to the Schrödinger equation. This evolution is deterministic and (complex) linear. This means that, once an initial state is given, the subsequent state is unambiguously determined for all future times. Moreover, if the time dependence of two states obeys the Schrödinger equation, any linear combination of them also satisfies that equation. The second principle of evolution, on the contrary, is stochastic. When the system is subject to a measurement process it happens that (i) the possible outcomes of such a measurement are the eigenvalues of the observable that is being measured and (ii) the state vector of the system, *immediately after* the measurement has been completed, becomes one of the eigenvectors of the observable measured, namely the one corresponding to the result actually observed, all the rest of the state vector being *instantaneously* annihilated (*reduction postulate*).

Photon physics has historically provided the testing ground for several foundational issues (for a recent review see ref.^1^). In particular a lot of work has been dedicated to the study of the so called EPR-B correlations and non-localitity issues implied by entanglement and violations of Bell’s inequalities. After the seminal work on entanglement and violation of Bell’s inequalities by^2 and 3^ most recently violation of Bell’s inequalities has been tested by T. Scheidl *et al*. in ref.^4^. Improving upon all existing experiments, the authors of ref.^4^ insure space-like separation of their measurement events in order to remove the possibility of transmitting any signal propagating at velocity ≤ *c* between the entangled particles. This Bell’s test was, amazingly, performed between the two Canary Islands La Palma and Tenerife, separated by 144 km. As a corollary of their Bell’s test one can argue that the *collapse* of the two-photon entangled state is *instantaneous*, as required by the second evolution postulate (see above) of the canonical formulation of quantum theory. More recently, the authors of ref.^5^ performed a Bell test using electrons separated by 1.3 km and obtained a loophole-free Bell inequality violation. In ref.^6^, a lower bound on the speed of nonlocal correlations in a Bell test has been set to be at least four orders of magnitude larger than the speed of light. Along the same line of research, the authors of ref.^7^ confirm the result of^6^ by also closing the locality and freedom-of choice lophooles over a distance of 16 km.

All this as far as two-photon correlations are considered, *i.e*. when two (entangled) photons *separately* hit their respective detector. When it comes to considering *single* photons, the situation is different. Actually, experiments using light at the single photon level have historically given crucial contributions to test the foundations of Quantum Mechanics. In a recent review paper (see ref.^1^) the main achievements have been described, with emphasis on two foundational themes, namely wave-particle duality and Bell nonlocality. During the 5th Solvay Conference in 1927 (see^8,9^) Einstein considered a single particle that, after diffraction at a pin-hole, encounters a “detection plate” (a photographic plate in the case of photons). To simplify the discussion to the essence, one could consider a single photon impinging on a 50:50 beamsplitter (BS), followed by two Single Photon Detectors (SPD), positioned along the two arms of propagation of the wave. Einstein stressed that only one of them can detect the particle, otherwise energy would not be conserved. But he was deeply concerned about the situation in which the two detection events are space-like separated, since this prevents any possible coordination among the detectors. The fact that a photon can be found only in one among a set of places is a manifestation of its particle character, while diffraction undoubtedly indicates that a wave character is also there. The two aspects of the behaviour of photons (more generally of microsopic particles) are made compatible by the statistical interpretation of the quantum mechanical state. The fact that nothing remains in the places where the photon is not found is conveyed by the reduction postulate. In experiments performed by splitting each photon of a beam this behaviour is named *antibunching*.

Actually, several experiments aimed at verifying antibunching have been devised and performed. Antibunching has been unambiguously proven by Clauser in ref.^10^: his experimental results, to a high degree of accuracy, contradict the predictions of any classical or semiclassical theory of light, confirming at the same time the prediction of Quantum Mechanics. A similar experiment was performed by Grangier *et al*. (see ref.^11^) who observed both antibunching and wave interference effects by a Mach-Zender interferometer with a visibility over 98%. More recently, heralded single photons have been used by Guerreiro *et al*. (see ref.^12^) to perform an antibunching experiment; the experimental set-up has been devised in such a way that the events consisting in the arrival of the photon wave packets at the detectors are space-like separated, and also in this case antibunching has been experimentally confirmed. Also, the experimental proof of nonlocal quantum state collapse for a single photon has been given in ref.^13^. The authors demonstrate the single particle nonlocal collapse by splitting a single photon between two laboratories and verifying that the choice of the measurement setup in one laboratory really causes the change of the local quantum state in the other laboratory by means of homodyne measurements. Moreover, experimental verification of antibunching was not limited to photons, but has been confirmed for other particles such as neutrons, as seen in ref.^14^.

What is missing in all experiments of this kind is a thorough control of the time of flight of the single photon, leaving the possibility that the coordination about which detector *clicks* (the detector at which the photon is going to *materialize*) could take a time.

The present paper concerns an experiment able to test whether the state vector collapse for a single particle, being not instantaneous, takes a time, in particular when the system is spatially delocalized. This is the first time that an experiment of this kind is performed using a single particle state instead of entangled EPR pairs: the use of single particle states is important to check whether the collapse process involving the actual displacement of the particle between the distant places requires a time, and could point out a possible difference with respect to EPR pairs, where only internal degrees of freedom are correlated like in EPR experiments.

## Experiment

### Conceptual Scheme

**Figure 1:**
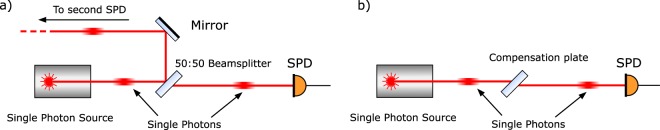
(**a**) Single photons on a 50:50 beamsplitter; in the forward direction a single photon detector (SPD) is positioned; a mirror sends the wave packet to the backward direction. (**b**) When the compensation plate replaces the beamsplitter, no superposition is prepared.

Let us consider the setup of Fig. 1a, where a beam of light impinges on a 50:50 BS. If the input is a single photon Fock state^15^, it is easy to show (the full calculation can be found in^15^) that the output is

1 $$|{\phi }_{out}\rangle =\frac{1}{\sqrt{2}}{(|1\rangle }_{f}+{\rm{i}}|1{\rangle }_{b}),$$

representing a single photon de-localized between the propagation directions forward and backward. The same superposition is not found for other type of inputs, in particular in the case of classical sources. If we consider a coherent state, as would be the case for a laser beam, of complex amplitude *α*

2 $$|\alpha \rangle ={e}^{-\frac{|\alpha {|}^{2}}{2}}\mathop{\sum }\limits_{n=0}^{\infty }\frac{{\alpha }^{n}}{\sqrt{n!}}|n\rangle ,$$

where |*n*〉 are Fock states^15^, then for a 50:50 BS the output becomes

3 $$|{\phi }_{out}\rangle ={|\frac{\alpha }{\sqrt{2}}\rangle }_{f}{|\frac{\alpha }{\sqrt{2}}\rangle }_{b}.$$

The output is therefore the factorized state of the coherent state ${|\frac{\alpha }{\sqrt{2}}\rangle }_{f}$ propagating forward and the coherent state ${|\frac{\alpha }{\sqrt{2}}\rangle }_{b}$ *independently* propagating backward. More in general, for a non-symmetrical BS the two states will be |*β*〉_*f*_ and |*γ*〉_*b*_, with |*β*|^2^ + |*γ*|^2^ = |*α*|^2^. Should the input be a pulse of thermal light (as in the case of lamps or LEDs), then one has to take into account that a single mode of thermal light (see for instance ref.^16^) can be described by the statistical operator

$$\rho =\int \,\frac{{d}^{2}\alpha }{\pi \langle n\rangle }\,\exp [-\frac{|\alpha {|}^{2}}{\langle n\rangle }]|\alpha \rangle \langle \alpha |,$$

where 〈*n*〉 is given by

$$\langle n\rangle ={[\exp (\frac{\hslash \omega }{kT})-1]}^{-1}$$

and *α* are coherent states as in Eq. (2). Being the input state a mixture of coherent states, also the output is a mixture of factorized forward and backward states.

According to Eq. (1), the quantum state of each single photon is coherently split as the superposition of “wave packet in the forward direction” and “wave packet in the backward direction”. Notice that at the BS no reduction of the quantum state takes place; this is a well established experimental fact, for instance in Mach-Zender interferometry, delayed-choice experiments and homodyne measuments^17,18^ Let us suppose first that the second detector is not there and that the wave packet can propagate freely to the backward direction. The initial state of the system “photon + detector” is given by

4 $$|{{\rm{\Psi }}}_{in}\rangle =\frac{1}{\sqrt{2}}{(|1\rangle }_{f}+{\rm{i}}|1{\rangle }_{b})|{D}_{0}\rangle ,$$

where |*D*_0_〉 represents the SPD in the state “ready”. With probability *p* = 1/2 the photon is detected by the SPD, and immediately after the measurement the quantum state of the system “photon + detector” is |*D*_+_〉, where |*D*_+_〉 represents the situation in which the photon has been absorbed and the detector fired; alternatively with probability *p* = 1/2, the photon is not detected by SPD and immediately after that the state of the system is |1〉_*b*_|*D*_0_〉. This is the description of what happens according to the reduction postulate. In formulae, taking into account Eq. (4):

5 $$|{\Psi }_{in}\rangle \to \{\begin{array}{ll}|1{\rangle }_{f}|{D}_{0}\rangle \to |{D}_{+}\rangle  & {p}_{f}=\frac{1}{2},\\ |1{\rangle }_{b}|{D}_{0}\rangle  & {p}_{b}=\frac{1}{2}.\end{array}$$

It is worth noticing that, in the case of a single photon, the reduction of the quantum state as described by Eq. (5) is a non-local “process”, the single photon being de-localized in both the forward and backward directions.

One may ask what “immediately after” means. The answer of standard quantum mechanics is “immediately after the wave packet in the forward direction has reached SPD and SPD has completed, with positive or negative result, the measurement process”, i.e. after the time of flight of the photon from the source to the detector, *T*, plus the reaction time of the detector, *δ*, that is of the order of nanoseconds or even picoseconds for nowadays devices. In a wide class of spontaneous collapse models, in which the quantum state collapse is a consequence of a modification of the Schrödinger equation effective at the macroscopic level (see for instance^19–22^), one must include in *δ* also the collapse time of the macroscopic pointer of SPD, a time that can be estimated to be of the order of a fraction of a picosecond.

The times considered up to now include the time of flight of the wave packet in the forward direction and the time demanded by processes taking place inside the detector. One can imagine as a possible scenario that some sort of process takes place between the wave packets, and that this process takes a time (collapse time), in particular when the wave packets are far apart. The imagined process would consist of some kind of coordination of the forward and backward wave packets in order to ensure that the photon “appears” only in the forward/backward positions (otherwise antibunching is not guaranteed and energy is not conserved), possibly taking place at a finite speed *c*′. Then, if the two paths are opposite to one another as indicated in Fig. 1, the required time would be of the order of Δ = 2*L*/*c*′, *L* being the distance between the BS and the SPD. Note that a (would–be) finite value of Δ is described neither in standard quantum mechanics nor in the collapse models mentioned above.

In the case in which a laser pulse is used instead of a single photon, taking into account eq. (2) the initial state of the system “photon + detector” can be written as

6 $$|{{\rm{\Psi }}}_{in}\rangle =|\alpha \rangle |{D}_{0}\rangle ={{\rm{e}}}^{-\frac{|\alpha {|}^{2}}{2}}\mathop{\sum }\limits_{n=0}^{\infty }\frac{{(\alpha {\hat{a}}_{in}^{\dagger })}^{n}}{n!}|0\rangle |{D}_{0}\rangle ,$$

and, by taking into account Eqs (3) and (6)

7 $$\begin{array}{ll}|\alpha \rangle |{D}_{0}\rangle  & \to |\gamma {\rangle }_{b}\mathop{\underbrace{|\beta {\rangle }_{f}|{D}_{0}\rangle }}\limits_{\,}\\  & \to (\begin{array}{ll}|\gamma {\rangle }_{b}\mathrm{|0}{\rangle }_{f}|{D}_{0}\rangle  & {p}_{0}={{\rm{e}}}^{-|{\beta }_{f}{|}^{2}},\\ |\gamma {\rangle }_{b}|ph{\rangle }_{f}|{D}_{0}\rangle \to |\gamma {\rangle }_{b}|{D}_{+}\rangle  & {p}_{ph}=1-{p}_{0}\mathrm{.}\end{array}\end{array}$$

In Eq. (7) |0〉_*f*_ and |*ph*〉_*f*_ are the normalized *vacuum* and *at least one photon* states in the forward direction; *p*_0_ and *p*_*ph*_ are the probability of finding the vacuum or at least one photon in the forward direction, respectively, dependent on the parameter *β*_*f*_.

It is worth noticing that in this case the collapse process involves the forward coherent state |*β*〉_*f*_ and the detector; hence the process is *local* and does not affect the distant coherent state |*γ*〉_*b*_, that is left unchanged. This remains true even if the input of the beam splitter is a weak coherent state, for which the average number of photons |*α*|^2^ is close or even smaller than one: the output is still a factorized state and its effects are intrinsically different from the ones of the single photon Fock state.

This in turn means that, in the assumption that the collapse of a *non-local* state requires a finite time in order to “coordinate” the distant parts of the quantum state, the finite collapse time is detectable *only* if a single photon state is used. Hence, if the initial state is a laser pulse the system is blind to a possible finite collapse time. The same is true for chaotic light, as thermal states can be expanded on the set of coherent states^16^, and would therefore result in a separable state after the BS. This is a very important point for this experiment: the fact that no additional delay would be observed using lasers implies that such an effect would be undetectable in time of flight measurements, in particular in LIDAR systems^23^ (even so called single-photon LIDAR systems work with Single Photon Detectors, but use laser pulses). Moreover, the delay due to the collapse would be undetectable by experiments that measure the time of flight of light produced by thermal sources, in particular distant astronomical sources^24^.

If the collapse time Δ is non-vanishing, it can be revealed by comparing the emission-detection times *T* + *δ* + Δ and *T* + *δ* for the setup of Fig. 1a (without or with the second SPD) in the case of a single photon state or a laser pulse as initial states, respectively.

As a cross check, one can also consider a setup like the one shown in Fig. 1b, where in place of the BS a Compensation Plate (CP) is inserted, in order to maintain the (small) delay the photon experiences when traveling through glass in both the experimental configurations.

### Experimental scheme

**Figure 2:**
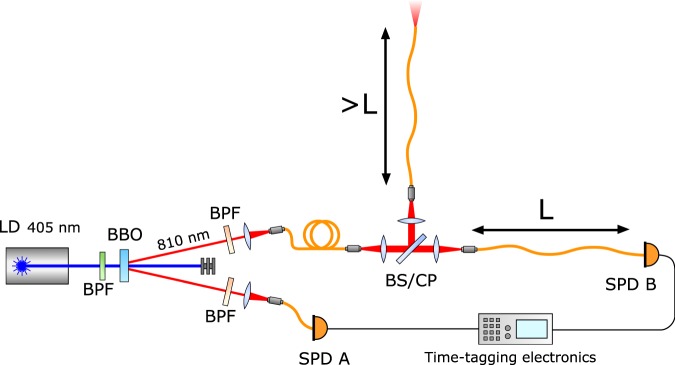
Experimental set-up. LD: Laser Diode; BBO: Beta-Barium Borate nonlinear crystal; BPF: 10 nm wide Band Pass Filter centered at 810 nm (fiber couplers) and 405 nm (laser diode); SPD: Single Photon Detector; BS: 50:50 beamsplitter; CP: Compensation Plate; the length L of the signal arm is varied between 2 and 20 m.

In order to experimentally test the hypothesis, we use a source of heralded single photons (ref.^25^), as schematized in Fig. 2. Such configuration allows to test the various scenarios taken into account in the previous section.

A nonlinear BBO crystal is used as the medium for Spontaneous Parametric Downconversion. The crystal is cut so that, for normal incidence of the pump beam, the phase matching condition is met when the down-converted photons are emitted inside a cone with half-opening angle of 3 degrees. The crystal emits pairs at a wavelength of 810 nm. The downconverted photons are collected and separately focused on multi-mode fibres. Single Photon Detectors (SPD A and SPD B) are used for detection and have an efficiency of around 5% at 810 nm and a time resolution of 35 ps. The electronic pulses output from the detectors are however slightly degraded while traveling through the long electronic cables, and the time resolution in the experiment is measured to be about 60 ps. Time-tagging electronics is used for coincidence counting.

The photon detected at SPD A heralds the single photon in the other arm and can be used as a time reference for the experiment; the time of click of SPD B can be compared when there is a BS, (i.e. the quantum state is split) or the CP (i.e. no choice of path).

When using the BS, comparison can also be made between the single photon non-separable state (as in Eq. 1) and the coherent state separable state (as in Eq. 3). This is performed by launching, before the BBO, coherent light produced by a pulsed supercontinuum laser through the same path of the downconverted photons.

### Experimental results

**Figure 3:**
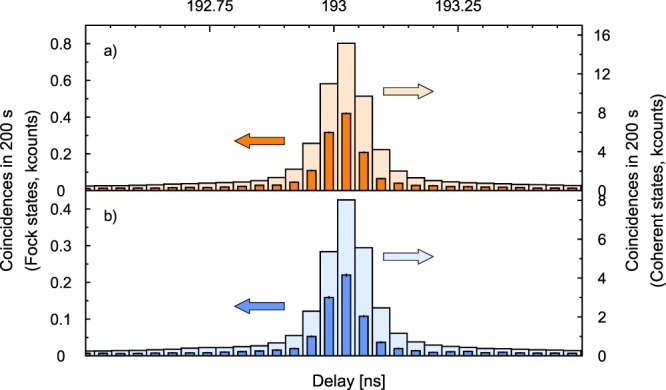
Coincidence histograms as a function of delay between the reaction times of the heralding and the distant SPDs when the 20 m fiber is connected (**a**) to a compensation plate or (**b**) to a beamsplitter. In both cases the lighter histogram is produced by coherent pulses while the dark one is produced by photon pairs. Integration time is 200 s in all cases.

The results obtained for the maximum length of the signal arm (20 m) are shown in Fig. 3, which shows the histogram of the coincidences between detectors A and B as a function of the time delay, both for quantum and coherent light. The histograms overlap perfectly and no relative delay is observed within the time resolution of the experiment.

**Figure 4:**
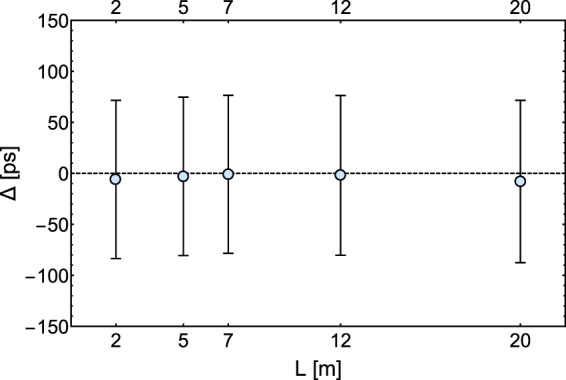
Difference between the time of arrival of the single photons and the coherent pulses (Δ), with the BS inserted along the path, as a function of the length of the fiber connected to the distant SPD. The error bars are the sum of the FWHM of the coincidence peaks of the single photon and coherent pulse signal.

The mean delay times for multiple lengths of the signal fiber L were determined by taking the peaks of the corresponding histograms, as well as their full width at half maximum. For every distance, Fig. 4 shows the difference between the mean delays for coherent and quantum light; no appreciable delay difference can be observed at any length.

## Discussion

The experimental data show clearly that the spatial collapse of the photon state takes a time shorter than the time resolution of our experiment, given the maximum available distance.

This allows us to put an upper limit on the collapse time, and hence on its speed. If a nonzero collapse time is actually present, its duration Δ has to be shorter than 60 ps, which is the time resolution of the experiment. The maximum length for the detector arm in the experiment was *L* = 20.04 m (±1 cm) so the maximum investigated distance between the two ends of the arms was $\sqrt{2}L=28.4\,{\rm{m}}$. We can therefore put a lower bound to the collapse speed *c*′ of 1550 times *c*, since any other less direct path followed by the wavefunction would result in a higher collapse speed. This value is about one order of magnitude smaller than the best current results on the speed of the *spooky action at a distance* of refs^6,7^, but complementary to that, giving information on the behaviour of a *single* delocalized particle rather than the correlations of internal degrees of freedom as in entangled EPR pairs.

In order to improve the present bound, an upgraded version of the experiment is being developed in which distances are increased up to the kilometric scale as done for EPR pairs^4,7^.

## Methods

To produce pairs by downconversion, we use a blue laser diode (Sanyo DL-LS5017) to pump the BBO crystal with 65 mW of continuous wave power at a wavelength of 405 nm. The beam is spectrally filtered by a 10 nm Band Pass Filter (BPF) centered at 405 nm. The BBO crystal is 1 mm thick and has a square 5 mm by 5 mm cross-section. The crystal is in sandwich configuration: two 0.5 mm crystals are stitched together rotated by 90 degrees. This configuration is useful to generate polarization entangled photon pairs, although in our experiment we are only interested in the generation of photon pairs and the subsequent heralding of single photons. Therefore, concerning conversion efficiency, the effective length of the crystal is 0.5 mm.

The downconverted photons are spectrally shaped by BPF’s with 10 nm bandwidth before they are focused to 60 *μ*m core diameter multi-mode fibres. The BS (Thorlabs BSW29R) and the CP (Thorlabs BCP44R) are 1 mm thick, 25 × 36 mm UV fused silica plates. The BS is metal coated, while the CP is anti-reflection coated.

The principal characteristics (quantum efficiency, response time, dead time) of the SPD’s (ID Quantique id100-MMF50-STD) were certified by the manufacturer and also experimentally verified before the measurements. Time-tagging electronics (Picoquant HydraHarp-400) is used for coincidence counting.

Note that the presented experimental scheme uses only information collected at SPD B. Indeed if the detector SPD B reacts to a single photon, a third detector on the other arm does not react; so the inclusion of a third detector does not convey any additional information on what is going on at SPD B, where all the observations used in the present experiment are performed. Hence the only experimental evidence that the collapse has truly ended is the reaction of SPD B.

## Data Availability

The dataset acquired during this work is available from the corresponding author on reasonable request.
